# Laser Linewidth Analysis and Filtering/Fitting Algorithms for Improved TDLAS-Based Optical Gas Sensor

**DOI:** 10.3390/s23115130

**Published:** 2023-05-27

**Authors:** Chen Tong, Chaotan Sima, Muqi Chen, Xiaohang Zhang, Tailin Li, Yan Ai, Ping Lu

**Affiliations:** 1 Next Generation Internet Access National Engineering Research Center, School of Optical and Electronic Information, Huazhong University of Science and Technology, Wuhan 430074, China; u201913960@hust.edu.cn (C.T.); m202273000@hust.edu.cn (M.C.); xiaohangzhang@hust.edu.cn (X.Z.); m202272654@hust.edu.cn (T.L.); m202172892@hust.edu.cn (Y.A.); pluriver@mail.hust.edu.cn (P.L.); 2 Wuhan OV Optical Networking Technology Co., Ltd., Wuhan 430073, China

**Keywords:** optical gas sensor, TDLAS, VMD-SG algorithm, laser linewidth, noise floor

## Abstract

Tunable Diode Laser Absorption Spectroscopy (TDLAS) has been widely applied in in situ and real-time monitoring of trace gas concentrations. In this paper, an advanced TDLAS-based optical gas sensing system with laser linewidth analysis and filtering/fitting algorithms is proposed and experimentally demonstrated. The linewidth of the laser pulse spectrum is innovatively considered and analyzed in the harmonic detection of the TDLAS model. The adaptive Variational Mode Decomposition-Savitzky Golay (VMD-SG) filtering algorithm is developed to process the raw data and could significantly eliminate the background noise variance by about 31% and signal jitters by about 12.5%. Furthermore, the Radial Basis Function (RBF) neural network is also incorporated and applied to improve the fitting accuracy of the gas sensor. Compared with traditional linear fitting or least squares method (LSM), the RBF neural network brings along the enhanced fitting accuracy within a large dynamic range, achieving an absolute error of below 50 ppmv (about 0.6%) for the maximum 8000 ppmv methane. The proposed technique in this paper is universal and compatible with TDLAS-based gas sensors without hardware modification, allowing direct improvement and optimization for current optical gas sensors.

## 1. Introduction

Tunable diode laser absorption spectroscopy (TDLAS) is a popular technical route in optical gas sensing. A narrow linewidth laser beam with good monochromaticity is used to scan the specific gas absorption line, then obtains the gas concentration according to the change of scanning laser power [1,2]. It has attracted great attention since initially proposed in the 1970s [3,4]. It is found that the sensing performance of TDLAS systems is affected by numerous factors, e.g., laser modulation, ambient environment, background noise, optical chamber, demodulation algorithm, etc. The literature has reported various efforts to achieve the enhancement, such as modified laser modulation, hollow core fibers as a gas chamber, optical dual-wavelength demodulation, and advanced filtering algorithms [5,6,7,8]. Among the literature, it was found that, in the modeling and simulation of the TDLAS technique, the laser source spectrum was merely simplified as straight lines or rectangular distribution approximately [1,2,3,4,5,6,7]. The influence of the laser spectral profile or laser linewidth was rarely considered and discussed. Therefore, it is worth exploring this effect for the laser source.

In the digital processing of TDLAS-based gas sensors, various filters, and denoising algorithms were employed [9,10,11,12]. In 2014, C. Zheng et al. used wavelet denoising to extract second harmonic signals from raw data and reduced the minimum detection limit (MDL) to 1 ppm [10]. Variational mode decomposition (VMD) algorithm was also employed, which could overcome the end effect of the empirical mode decomposition (EMD) algorithm and the challenges in screening iteration of the eigenmode function and modal aliasing [11,12]. The Savitzky-Golay (SG) filter was also introduced to deal with high-frequency data. In 2020, T. P. Banjade et al. combined the VMD algorithm with an SG filter to monitor earthquakes [13]. In 2021, H. Yang et al. used the VMD-SG algorithm to suppress the ship-radiated noise [14]. Hence, it is interesting to explore the VMD-SG algorithm for signal processing in the TDLAS-based gas sensors.

In this paper, we proposed and experimentally demonstrated an advanced TDLAS-based optical gas sensor with laser linewidth analysis and improved filtering/fitting algorithms. The linewidth of the laser pulse spectrum is innovatively considered and analyzed in the harmonic detection of the TDLAS model. In the experimental setup, the adaptive VMD-SG filtering algorithm is developed and introduced for the raw data processing and could significantly eliminate the background noise and signal jitters, compared to original signals or using a conventional wavelet algorithm. Furthermore, the Radial Basis Function (RBF) neural network is also incorporated and applied to improve the fitting accuracy of the gas concentration. Compared with traditional linear fitting and least squares means (LSM) fitting, the RBF neural network brings along enhanced fitting accuracy within a large dynamic range, obtaining an absolute error of below 50 ppmv for the maximum 8000 ppmv methane. Hence, the proposed technique in this paper is universal and compatible with TDLAS-based gas sensing without hardware modification, allowing direct and effective improvement and optimization for current optical gas sensors.

## 2. Operation Principle

### 2.1. Laser Spectrum and Linewidth

For diode lasers used as optical sources in TDLAS, the output spectrum normally exhibits a narrow linewidth with certain broadening. The linewidth broadening caused by different physical broadening mechanisms is varied, such as natural broadening, collision broadening, and Doppler broadening. In practice, the spectral lines of gases are often affected by combinations of these broadening mechanisms, while collision broadening, and Doppler broadening are basically considered in this work.

The distributed feedback (DFB) laser is widely used in TDLAS systems because of its narrow linewidth, tunability, and low cost [15]. The output spectrum of DFB lasers is normally considered a Gaussian-like distribution. Generally, the linewidth of the laser is defined as the wavelength coverage width (Δλ) when the laser intensity decreases to half of the maximum. When the light intensity drops to half of the maximum value, this wavelength range is also called the full width at half maximum (FWHM). These definitions are utilized in the following modeling and simulation.

### 2.2. TDLAS with Harmonic Detection

The theoretical basis of TDLAS is the Beer–Lambert law [16], giving the relationship between the absorbance, path length, and concentration of light-absorbing substances. If the incident light intensity is *I*_0_ and the attenuated outgoing light is *I_t_*, as shown in Formula (1), then:

(1) $$I_{t}=I_{0}e^{-\alpha \left(\nu \right)Cl}$$

where, *α* is the absorption coefficient or extinction coefficient, which is the unique property of matter; *C* is the concentration, and *l* is the optical path.

**Figure 1:**
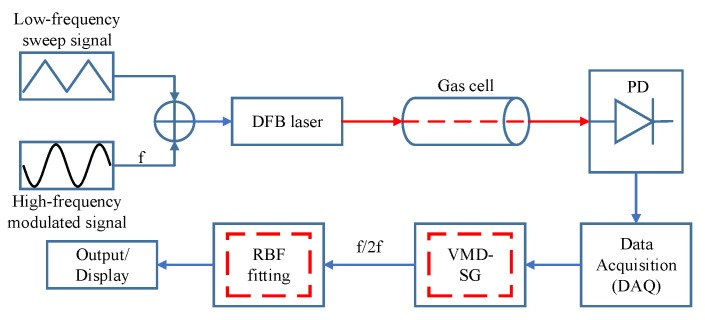
Block diagram of the TDLAS with the harmonic detection technique. Red dashed boxes show the proposed filtering and fitting algorithms. DFB: distributed feedback. PD: Photodiode.

TDLAS modulates the output wavelength of the laser by using the characteristic that the output wavelength of the laser changes with the injection current and scans the specific gas absorption spectrum with a narrow linewidth laser beam with good monochromaticity. The wavelength modulation spectroscopy (WMS) with harmonic detection is often employed in TDLAS. The first or second harmonics based on WMS are generated and detected. The TDLAS system diagram is schematically shown in Figure 1 and the red dashed boxes indicate the proposed VMD-SG filtering algorithms and RBF fitting algorithms in this paper.

## 3. TDLAS System and Laser Linewidth Analysis

### 3.1. Modelling and Simulation

**Figure 2:**
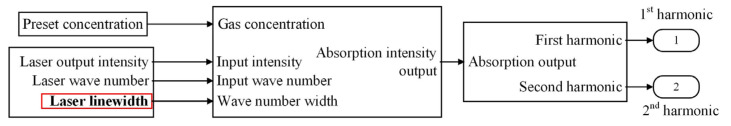
Simulation block diagram of the TDLAS-based gas sensor. The red box indicates the parameter for laser linewidth.

Modeling and simulation of the TDLAS sensor was established by using the Simulink software, mainly including the laser modulation module, the gas absorption module, and data processing module, as shown in Figure 2. The laser modulation module generates the modulated driving signals for the diode laser. In the gas absorption module, the optical path and gas absorption coefficient are preset, and the incident laser beam passes through the gas chamber. The absorbed laser beam is detected and converted by the photodiode. The electric signal from the photodiode is analyzed and demodulated through the signal processing model to obtain the first and second harmonics. Here, the laser spectrum and linewidth are innovatively considered and incorporated into the simulation, as labeled in the red box in Figure 2.

### 3.2. Laser Linewidth Analysis with Gas Absorption

In the simulation and modeling of TDLAS, the laser source was often regarded as an ideal straight line or rectangular spectrum, and the influence of laser spectral shape and linewidth were ignored in the literature. Therefore, the laser spectral shape and linewidth are introduced into the TDLAS system in this paper to explore the influence on harmonics.

The normalized Gaussian spectrum is regularly used to approximate the laser spectral broadening, as shown in the formula:

(2) $$g_{j}\left(\nu -\nu_{0}\right)=\frac{2}{\Delta \nu_{i}}\sqrt{\frac{\ln2}{\pi }}\exp\left(\frac{-4\ln2{\left(\nu -\nu_{0}\right)}^{2}}{\Delta \nu_{i}^{2}}\right)$$

(3) $$\Delta \nu_{i}=\nu_{0}\sqrt{\frac{8\ln2kT}{Mc^{2}}}$$

where Δ*v_i_* is the linewidth of the Gaussian spectrum function and *M* is the molar mass of the light-absorbing material.

**Figure 3:**
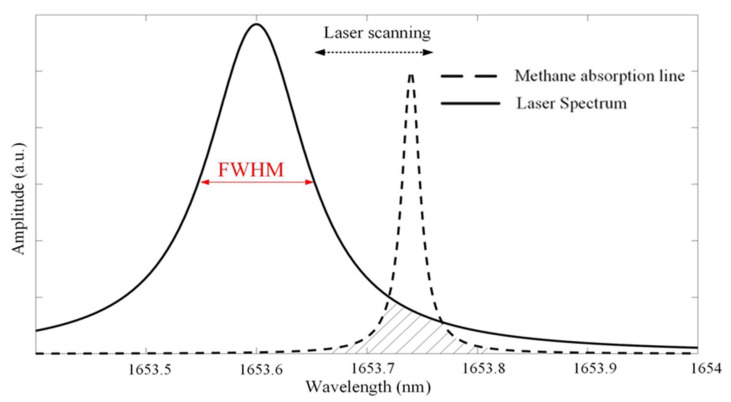
Schematic diagram of the interaction between laser spectrum and methane absorption line.

The interaction between the laser spectrum and gas absorption is schematically shown in Figure 3. The FWHM of the laser is assumed to be varied around 0.1 nm, and the peak intensity remains constant for simplicity. The absorption line of methane is selected at around 1653.7 nm. The laser is scanned and swept across the gas absorption line. The overlapped and shaded area in Figure 3 indicates that optical gas absorption occurs within this area and leads to the corresponding laser intensity attenuation. The convolution of these two spectral functions is used to calculate the power attenuation:

(4) $$S_{g}\left(\nu \right)=g_{L}\left(\nu -\nu_{0}\right)*g_{i}\left(\nu -\nu_{0}\right)$$

**Figure 4:**
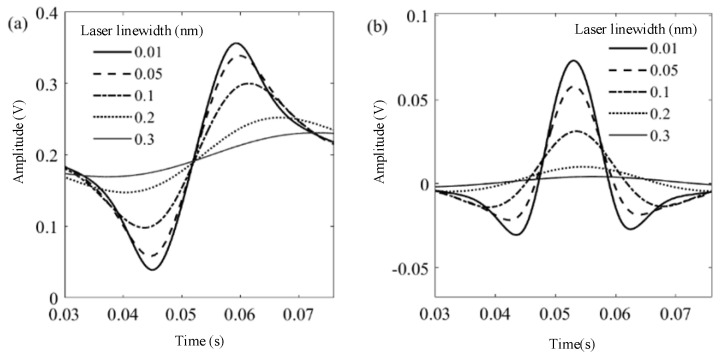
Influence of laser linewidth on harmonics: (**a**) first harmonics; (**b**) second harmonics.

In the modeling, the parameters are set as follows temperature of 296 K, gas pressure of 1 atm, methane concentration of 0.1% in volume, and the optical path of 10 cm. The influence of laser linewidth on the harmonic waveform in the TDLAS system is analyzed and shown in Figure 4. Results show that, with the increase of laser linewidth (from 0.01 nm to 0.3 nm), peak-to-peak values of first harmonics and peak values of second harmonics are both gradually decreasing. These essential values of harmonics are proportional to the gas sensitivity. Hence, it is concluded in the simulation that the use of a narrow linewidth laser could enhance the sensitivity and accuracy of the TDLAS-based gas sensors.

## 4. Experimental Setup and Filtering/Fitting Algorithms

**Figure 5:**
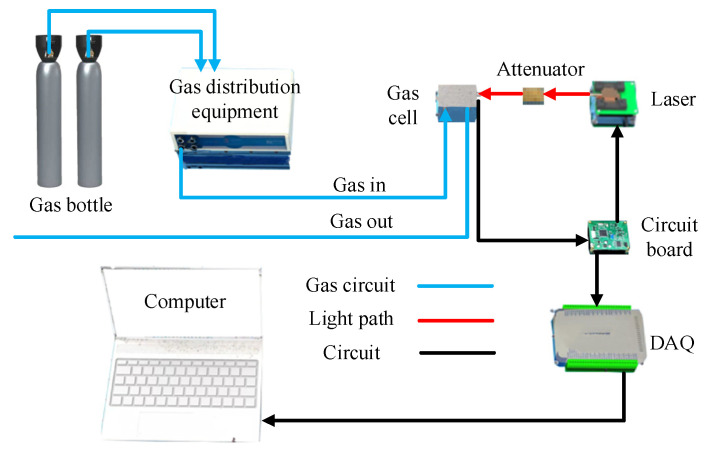
Experimental setup of the TDLAS-based methane sensor.

The experimental setup of the TDLAS-based gas sensor is schematically shown in Figure 5. An STM32 chip functions as a waveform generator to generate a 5 Hz triangular scanning signal and 1 kHz sinusoidal modulation signal, and the laser driver. An ADN8834 chip was used for TEC control to stabilize the output intensity and central wavelength of the DFB laser. The modulated laser beam enters into the gas cell and is then received and converted by the photodiode (PD) into a voltage signal. It is then uploaded to the laptop by the data acquisition card for subsequent analysis and digital processing. A data acquisition card (SMACQ, USB-5311) collected and digitalized the required harmonics. Gas distribution equipment (MCQ, GB100) is used for gas mixtures with different concentrations. In the MCQ GB100, the input flows are measured by an airflow sensor and regulated through a proportional valve that establishes the proper mass flow through each channel. Gas flow may be set through official software, and the actual flow value may be continuously monitored in the software interface. The MCQ GB100 could mix and blend up to three channels for non-aggressive gases.

### 4.1. VMD-SG Filtering Algorithm

In view of the limited SNR due to low concentration or environmental interference, this paper proposed the improved VMD-SG algorithm to reduce the signal noise floor. It is acknowledged that the wavelet denoising algorithm is frequently employed in signal processing. Hence, it is also considered in this work for comparison.

VMD originated from EMD and overcomes the end effect of the EMD algorithm, and the difficulty in determining the stopping standard of screening iteration of eigenmode function and modal aliasing. It has an adaptive and non-recursive modal variation for non-stationary and nonlinear signals. Additionally, the SG filter could be combined to deal with high-frequency data with limited SNR. The method of combining the VMD algorithm with the SG filter to reduce noise has been reported in earthquake monitoring and ship radiation denoising [10,11]. Here, this VMD-SG algorithm is improved adaptively and introduced to TDLAS for raw data processing.

**Figure 6:**
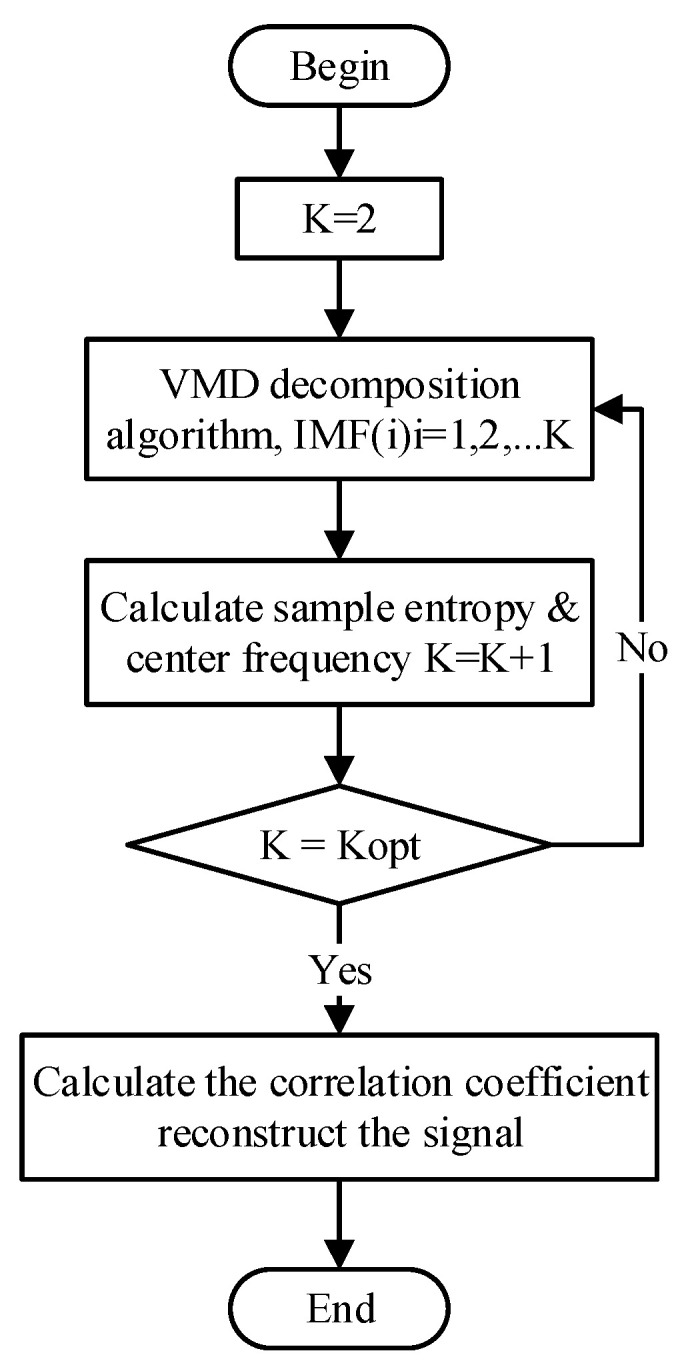
Flow chart of the adaptive VMD-SG algorithm.

Conventionally, the number of modal components K in VMD needs to be adjusted manually, and it is physically challenging to balance the time consumption and accuracy in VMD. When the value of K is extended, the difference between the center frequencies of adjacent modal components is small, which may cause modal repetition and increase the calculation time. If the value of K is slight, the signal cannot be completely decomposed, which will affect the accuracy of subsequent detection. Considering the major variation of different modes lies in the central frequency and its correlation with the original signal, it is feasible to realize the automatic adjustment of K by using the modal sample entropy and central frequency to represent and feedback the sequence complexity. Therefore, this work proposes the adaptive process of K value in the VMD-SG algorithm, as shown in Figure 6. This algorithm has a certain improvement effect on the “slow variable signal” with serious noise influence. When the signal changes rapidly (or with more effective high-frequency components), this algorithm may lead to the opposite effect on the signal. This algorithm is applicable to the signal with serious noise influence, and the signal amplitude is small in a certain period of time.

**Figure 7:**
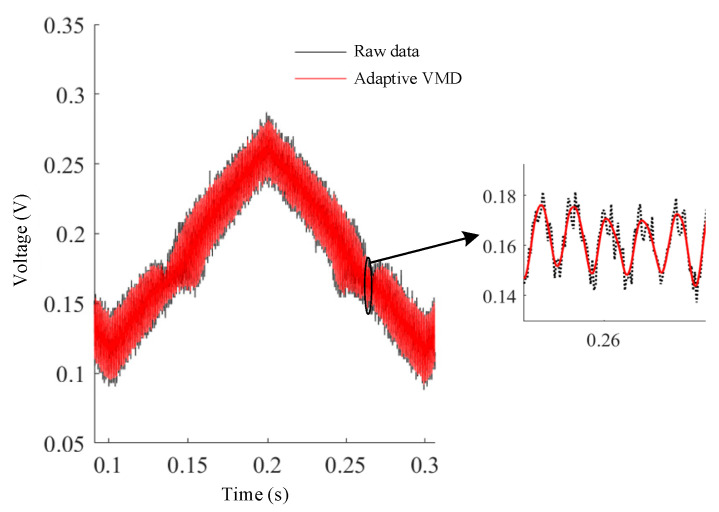
Processed data using an adaptive VMD algorithm from the photodiode.

**Figure 8:**
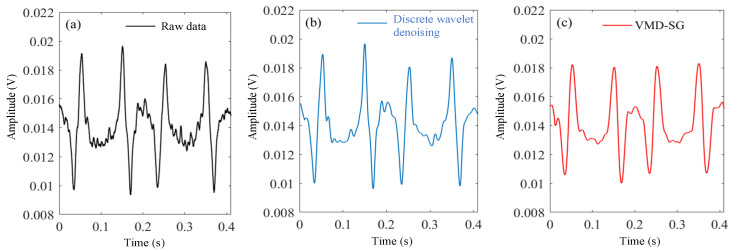
First harmonics: (**a**) Original raw data. (**b**) Discrete wavelet denoising. (**c**) Adaptive VMD-SG.

By using the adaptive VMD in the experiment, the decomposed and reconstructed signal from the PD is shown in Figure 7. It is obvious that the signal noise has been effectively suppressed. The reconstructed waveform is phase-locked to obtain harmonics. In order to further improve the detection accuracy, the harmonics are further processed by the SG filter. Figure 8 shows the first harmonics signals in the experiments, and the adaptive VMD-SG filtering algorithm allows stable and smooth signal processing beyond the unfiltered original raw data or those using the discrete wavelet denoising algorithm.

**Figure 9:**
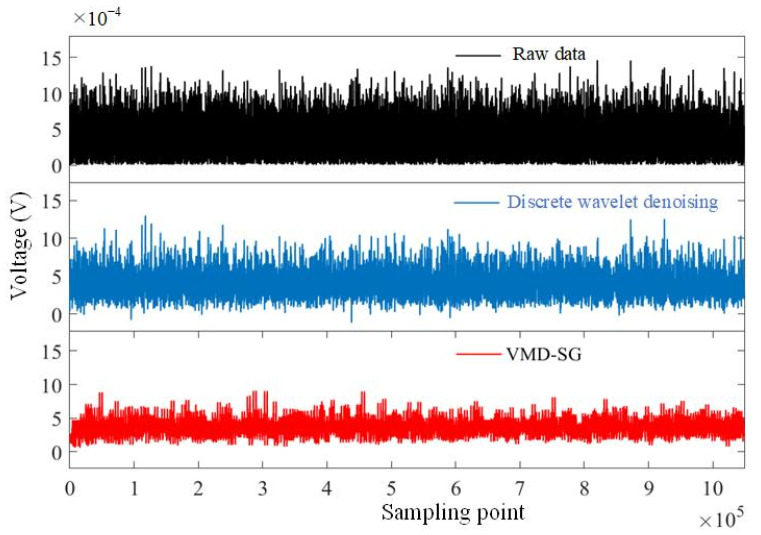
Comparison of the noise floors in the TDLAS sensor with different filters.

By using the above-mentioned adaptive VMD-SG algorithm, the gas sensing experiment is carried out with four concentrations of methane: pure nitrogen (methane free), 800 ppmv, 1000 ppmv, and 1200 ppmv. The background noise signals (in μV level) of the TDLAS system were measured for 30 min, as shown in Figure 9. The average noise floor standard deviation of the original raw data, using discrete wavelet denoising and using the proposed VMD-SG algorithm, are measured to be around 213.62 μV, 161.52 μV, and 113.32 μV, respectively. Compared with wavelet denoising, this algorithm can improve the noise floor by about 31%. It is concluded that the noise floor could be significantly reduced with the developed VMD-SG algorithm.

**Figure 10:**
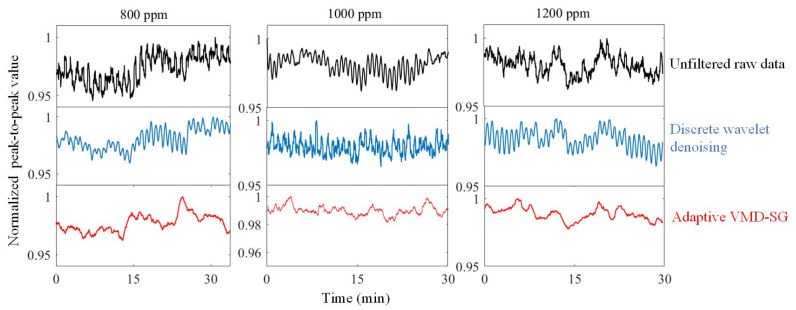
Normalized peak-to-peak value of first harmonics with different concentrations.

The stability of the gas sensor is also demonstrated experimentally. Figure 10 shows the sensing results using different filter algorithms with methane concentrations of 800 ppm, 1000 ppm, and 1200 ppm. Taking 800 ppmv for example, the original raw data of the peak-to-peak value of first harmonics fluctuate within about 5.5% region (94.5% to 100%). If using wavelet denoising, it is improved to about 4.5% in fluctuation. While using the adaptive VMD-SG algorithm, it is further improved to below 4%. Specially, the signal jitter has been obviously eliminated. Hence, the developed adaptive VMD-SG algorithm could improve the noise floor and the signal fluctuation, leading to the benefits of enhanced SNR and stability.

### 4.2. RBF Neural Network Fitting

In the TDLAS-based gas sensors, it is always required to accurately derive the generated harmonics with the gas concentration. Normally, it is calibrated by a fitting curve between the harmonics data and standard gas concertation. Due to the system noise and unexpected disturbance, this relationship rarely forms an ideal linear equation or simple nonlinear functions. Generally, the least square method (LSM) fitting is often used to obtain the concentration inversion relationship. This paper proposes an RBF neural network algorithm instead of LSM to achieve a more accurate fitting.

**Figure 11:**
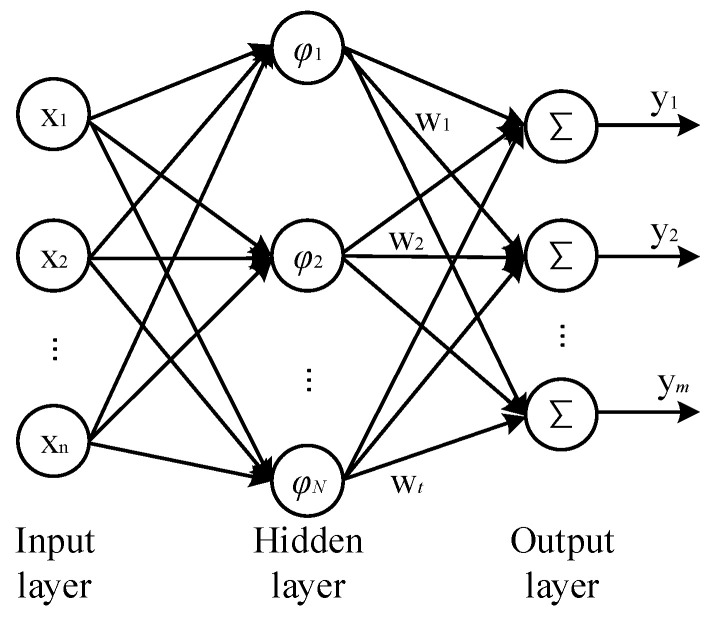
Topology of kernel-based RBF neural network.

The RBF neural network Is a feedforward neural network with local approximation ability. The network is generally composed of three layers: the input layer composed of input data, the hidden layer, and the output layer of nonlinear processing neurons, as shown in Figure 11.

**Figure 12:**
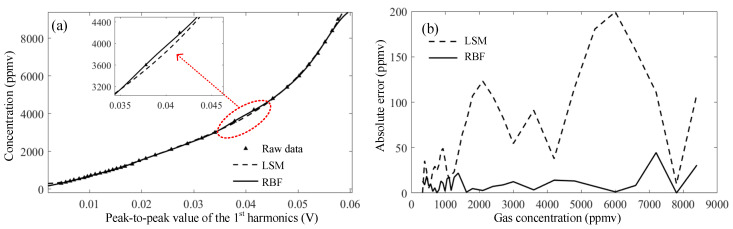
Concentration fitting and inversion. (**a**) Different fitting curves; (**b**) absolute sensing errors.

In order to verify the accuracy, 68 groups of gases with different concentrations were prepared by using the gas blender. A total of 34 groups were selected as training data, while the other 34 groups as test data. The raw data and the LSM fitting and RBF neural network fitting are illustrated for comparison, as shown in Figure 12a. The insert in Figure 12a presents the enlarged fitting effect around 4000 ppmv. Figure 12b shows the absolute error between the retrieved value and the true concentration. The absolute error of LSM fitting rises significantly when the gas concentration goes beyond 2000 ppmv and reaches 200 ppmv when the gas concentration is around 6000 ppmv. When using the RBF fitting, it shows favorable and continuous fitting accuracy and the absolute error does not exceed 50 ppmv across the full range of 0~8000 ppmv.

Table 1 exhibits the parameters of the fitting curves using LSM and RBF. The sum of squares of errors (SSE) and the root-mean-square error (RMSE) using RBF are both significantly reduced to 0.065% and 7.6%, respectively. The R-square in RBF fitting reaches 0.9999, indicating the nearly ideal fitting results. Hence, it is concluded that the RBF neural network fitting for this TDLAS-based gas sensor has a great accurate correlation with the true concentration. To be noted, at low concentrations, the absolute error is relatively large and could be further improved. In addition, this experiment is based on the premise of large-volume data fitting. If the data volume is reduced, the advantage of RBF fitting may be limited.

## 5. Conclusions

In this paper, the advanced TDLAS-based optical gas sensor was proposed and experimentally demonstrated. The spectral linewidth of the laser source was first considered and analyzed, showing improved sensitivity with narrow linewidth. The adaptive VMD-SG filtering algorithm was developed for the raw data processing to reduce the noise floor from 213 μV to about 113 μV and simultaneously enhance the stability of the system. By using this VMD-SG algorithm, the high-frequency signal jitter was eliminated to about 1% in the experiment. Furthermore, the RBF neural network was also investigated to improve the gas concentration fitting and inversion. Compared to the conventional LSM fitting, it allows favorable inversion accuracy and reduced fitting errors (below 50 ppmv) with concentrations up to 8000 ppmv. The proposed techniques in this paper are universal and compatible with TDLAS-based gas sensing without any hardware modification, allowing straightforward optimization for current optical gas sensors.

## Figures and Tables

**Table 1:** Parameters of fitting curves using LSM and RBF.

Parameter	LSM Fitting	RBF Fitting
SSE	289,600	1877.3
RMSE	98.24	7.4307
R-square	0.9987	0.9999

## Data Availability

Not applicable.
